# Neuroprotective Effect of Flavonoid Agathisflavone in the Ex Vivo Cerebellar Slice Neonatal Ischemia

**DOI:** 10.3390/molecules29174159

**Published:** 2024-09-02

**Authors:** Rodrigo Barreto Carreira, Cleonice Creusa dos Santos, Juciele Valeria Ribeiro de Oliveira, Victor Diogenes Amaral da Silva, Jorge Maurício David, Arthur Morgan Butt, Silvia Lima Costa

**Affiliations:** 1 Laboratory of Neurochemistry and Cellular Biology, Institute of Health Sciences, Federal University of Bahia, Av. Reitor Miguel Calmon S/N, Salvador 40231-300, BA, Brazil; rodrigo.carreira@ufba.br (R.B.C.); cleonicemev@gmail.com (C.C.d.S.); juciele.valeria@ufba.br (J.V.R.d.O.); vdsilva@ufba.br (V.D.A.d.S.); 2 Department of General and Inorganic Chemistry, Institute of Chemistry, University Federal da Bahia, Salvador 40170-110, BA, Brazil; jmdavid@ufba.br; 3 School of Medicine, Pharmacy and Biomedical Sciences, University of Portsmouth, Portsmouth PO1 2DT, UK; 4 National Institute of Translational Neuroscience (INNT), Rio de Janeiro 21941-902, RJ, Brazil

**Keywords:** Ischemia, agathisflavone, oligodendrocyte, astrocyte, neuroprotection

## Abstract

Agathisflavone is a flavonoid that exhibits anti-inflammatory and anti-oxidative properties. Here, we investigated the neuroprotective effects of agathisflavone on central nervous system (CNS) neurons and glia in the cerebellar slice ex vivo model of neonatal ischemia. Cerebellar slices from neonatal mice, in which glial fibrillary acidic protein (GFAP) and SOX10 drive expression of enhanced green fluorescent protein (EGFP), were used to identify astrocytes and oligodendrocytes, respectively. Agathisflavone (10 μM) was administered preventively for 60 min before inducing ischemia by oxygen and glucose deprivation (OGD) for 60 min and compared to controls maintained in normal oxygen and glucose (OGN). The density of SOX-10^+^ oligodendrocyte lineage cells and NG2 immunopositive oligodendrocyte progenitor cells (OPCs) were not altered in OGD, but it resulted in significant oligodendroglial cell atrophy marked by the retraction of their processes, and this was prevented by agathisflavone. OGD caused marked axonal demyelination, determined by myelin basic protein (MBP) and neurofilament (NF70) immunofluorescence, and this was blocked by agathisflavone preventative treatment. OGD also resulted in astrocyte reactivity, exhibited by increased GFAP-EGFP fluorescence and decreased expression of glutamate synthetase (GS), and this was prevented by agathisflavone pretreatment. In addition, agathisflavone protected Purkinje neurons from ischemic damage, assessed by calbindin (CB) immunofluorescence. The results demonstrate that agathisflavone protects neuronal and myelin integrity in ischemia, which is associated with the modulation of glial responses in the face of ischemic damage.

## 1. Introduction

Cerebral ischemia is a major consequence of stroke [1], which is a significant cause of morbidity and mortality worldwide [2]. Ischemia results in brain tissue damage, neuronal cell death, and cerebral infarction and is a key factor in multiple neuropathologies, including perinatal ischemic stroke, cerebral palsy, multiple sclerosis, neuroinfection, and traumatic brain injury [3,4].

Following ischemic damage, in the initial phase, cells that are compromised release cytokines, including transformation growth factor (TGF-*α*), IL-1, IL-6, and kallikrein-related peptidase 6 [5]. This action triggers the astroglial response, leading to the development of a perilesional barrier (*glia limitans peilaesiones)* encircling the damaged tissue [6], as well as a rapid activation of microglia, leading to the release of proinflammatory cytokines such as TNF-*α*, IL-1beta, and IL-6, thereby contributing to the escalation of the inflammatory response [7]. Oligodendrocytes, the central nervous system myelinating cells, are particularly vulnerable to ischemic damage [8,9,10], and this is recognized as a crucial element contributing to myelin loss and axonal degeneration following ischemia [11,12]. Multiple factors are involved in ischemic damage, including oxygen–glucose deprivation (OGD), which has been shown to induce myelin damage, leading to white matter injury [13]. OGD has been used extensively as an experimental model for inducing conditions that simulate ischemic events and impact oligodendrocytes and oligodendrocyte precursor cells (OPCs), affecting their survival, proliferation, and differentiation, with calcium-permeable AMPA/kainate and NMDA receptors playing an important role in this damage [14,15].

Flavonoids are increasingly recognized as having potential neuroprotective therapeutic value. Flavonoids are secondary metabolites of plants [16], and the human diet contains a wide range of phytochemical flavonoids [17,18], whose consumption is associated with the improvement in neurodegenerative diseases such as dementia and other neuronal pathological conditions [19]. Agathisflavone (bis-apigenin) is a flavonoid that has been purified from plants of different genera, such as *Ouratea giligiana*, *Rhus dentata*, *Anacardium occidentale* (cashew tree), and *Poincianella pyramidalis* (catingueira) [20]. Agathisflavone has demonstrated anti-neuroinflammatory neuroprotective properties associated with the regulation of microglial responses, protecting neurons against neurotoxicity induced by inflammatory stimuli and endogenous neurotoxins [21,22]. Moreover, our previous studies provided evidence that agathisflavone promotes myelination following the induction of demyelinating damage with lysolecithin in organotypic cerebellar slices [23,24].

In this context, the aim of this study is to characterize the neuroprotection mechanisms of the flavonoid agathisflavone (bis-apigenin) related to oligodendroglial plasticity and glial responses in an ex vivo model of ischemic damage. Here, we demonstrate that agathisflavone prevents oligodendroglial damage and myelin loss associated with altered astrocyte reactivity in the OGD model of ischemia in neonatal cerebellar slices. These results underscore the promising pharmacological prospects of agathisflavone as an adjunct therapy in protecting oligodendrocytes and myelin against ischemic damage.

## 2. Results

### 2.1. The Flavonoid Agathisflavone Prevented the Loss of Oligodendrocyte Processes

**Figure 1:**
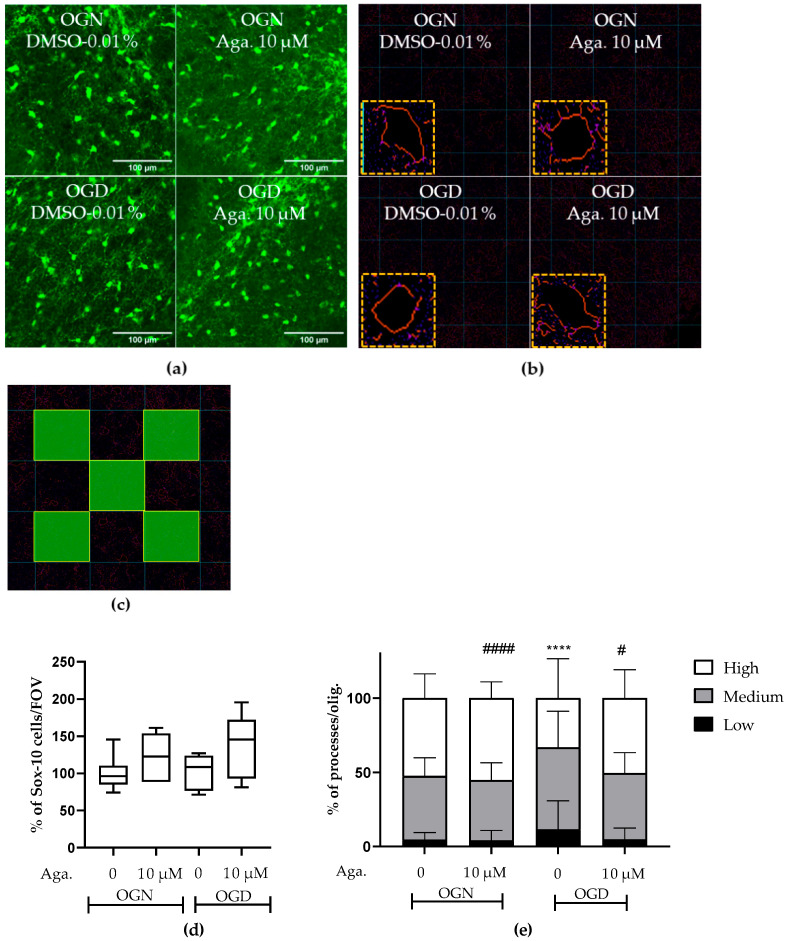
Assessment of density and morphology of oligodendrocytes in cerebellar slices subjected to ischemia. Organotypic culture of sagittal slices of cerebellum, 200 μm thick, from transgenic mice with green fluorescent protein reporter of the SOX-10 gene, aged between 8 and 12 d. (**a**) Images of oligodendrocytes obtained with a confocal microscope with a 20× magnification and 1.5× zoom objective, the scale bar represents 100 μm. (**b**) Representative images of the skeletonization of the delimitation of the cell body and processes of SOX-10 cells and highlighting skeletonized cells and respective cell body processes. (**c**) Representative scheme of the process quantification area in SOX-10 cells. The total counting area in each image was 25,000 μm^2^. (**d**) Representative box-plot graphs of the density of SOX-10 cells in the granular layer in the constant field of view (FOV) of 286.43 μm × 286.43 μm. Data represents the analysis of 2 to 3 images per slice obtained from 3 to 4 cerebellar slices of 3 to 4 animals. The percentage was calculated relative to OGN dimethyl sulfoxide (DMSO) 0.01% and is expressed as median, interquartile range, minimum, and maximum values; statistical significance was assessed by analysis of variance, with the Kruskal–Wallis statistical test. (**e**) Graphs of the percentage proportion of the distribution of cells with low (0–4), medium (5–9), and high (>9) processes per SOX-10 cell. Data is expressed as the mean and standard deviation of 2 to 3 images per slice obtained from 3 to 4 cerebellar slices of 3 to 4 animals; statistical significance was assessed with the *χ*^2^ statistical test comparing the proportions obtained in the different conditions, with the expected proportions, taking as a reference the proportion obtained in OGN-DMSO. **** *p* < 0.0001; or OGD-DMSO 0.01%: # *p* < 0.05 and #### *p* < 0.0001.

The concentration of agathisflavone (10 μM) was selected based on our previous published work that this concentration was the most efficacious in protecting oligodendrocytes and myelin in the lysolecithin model of demyelination in cerebellar slice cultures [24,25]. In the CNS, the SOX-10 gene is expressed throughout the oligodendroglial lineage and is an essential component of the transcriptional regulatory network of myelination [26,27], and expression of SOX10-EGFP enabled identification of oligodendrocytes and their precursors in the cerebellar slices following the different treatments (Figure 1). It was observed that in the slices treated with agathisflavone (10 μM), there was no change in the density of SOX-10 cells (Figure 1a,d). It was also found that 1 h of OGD did not cause a significant reduction in the number of oligodendrocytes compared to the OGN control group in the granular layer (Figure 1a,d); as observed in the OGN condition, agathisflavone (10 μM) in OGD did not induce changes in the density of SOX-10 cells.

It has been reported that ischemia can induce a retraction of oligodendrocyte processes, decreasing their myelination capacity [9,10], and so we evaluated the effect of ischemia and agathisflavone on the morphology of SOX10-EGFP+ oligodendroglia. The processes of oligodendrocytes emitting from the cell body were evaluated (Figure 1b,e) in images of the granular layer in SOX-10 cells. In cerebellar slices treated with agathisflavone, there was no difference in SOX-10 cells regarding the number of processes compared to those in OGN (Figure 1e). When the slices were subjected to 1 h of OGD, it was observed that the morphology of SOX-10 cells was different compared to OGN. In OGD, there was an increase in cells with low processes and a reduction in cells with many processes, revealing a retraction of oligodendroglial cell processes in ischemia (Figure 1e). In OGD cultures exposed to agathisflavone, the regression of processes seen in OGD slices was not observed (Figure 1e).

### 2.2. Agathisflavone Prevented White Matter Loss in Cerebellar Slices Subjected to Ischemic Damage

**Figure 2:**
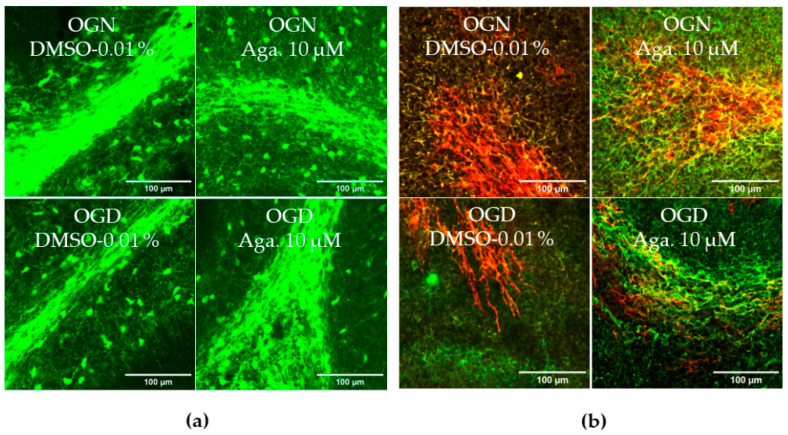

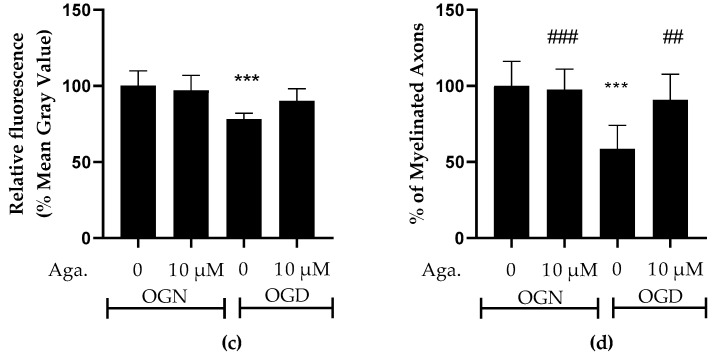
Effects of agathisflavone on cerebellar white matter following ischemic damage. (**a**) Organotypic culture of 200 μm thick cerebellar slices in sagittal position from mice aged 8 to 12 d, transgenic with green fluorescent protein reporter of the SOX-10 gene. Images of the white matter were obtained using a confocal microscope with a 20× objective and 1.5× zoom. (**b**) Organotypic culture of cerebellar slices in the sagittal plane, 200 μm thick, from C57BL/6 mice aged between 8 and 12 d. Images of cerebellar slices stained with MBP (red), NF70 (green), and colocalization of MBP and NF70 (yellow) were obtained with a confocal microscope with a 20× objective and 1.5× zoom. (**c**) Representative bar graphs of SOX-10 expression in the white matter region of interest (ROI). The percentage was calculated relative to OGN DMSO 0.01%; the data represent the analysis of 2 to 3 images per slice obtained from 3 to 4 cerebellar slices of 3 to 4 animals. The fluorescence was evaluated by the average gray value and is expressed as the mean and standard deviation; statistical significance was assessed using an analysis of variance ANOVA, *F (3*, *20)* = *8.314*, followed by Tukey’s multiple comparison post-test. The *p*-value is represented by *** *p* < 0.001 compared to the control group (OGN-DMSO 0.01%). (**d**) Data represent the percentage of myelinated axons in the granular layer and white matter in the constant field of view (FOV) of 289.53 μm × 289.53 μm, evaluated by correlation with the Pearson’s coefficient between the MBP staining channels and NF70 and are expressed as mean and standard deviation of 2 to 3 images per slice from 3 to 4 cerebellar slices of 3 to 4 animals and the percentage was calculated relative to 0.01% OGN DMSO; statistical significance was assessed using an analysis of variance ANOVA, *F (3*, *24) = 9.500*, followed by Tukey’s multiple comparison post-test. The *p*-value is represented by *** *p* < 0.001 compared to the control group (OGN-DMSO 0.01%), and ## *p* < 0.01, ### *p* < 0.001 compared to ischemic damage (OGD-DMSO 0.01%).

Taking into account the reduction of processes in oligodendrocytes induced by ischemia, it was evaluated whether this affected the cerebellar white matter and myelination (Figure 2). To this end, the intensity of SOX10-EGFP reporter fluorescence was quantified (Figure 2a,c). It should be noted that the EGFP reporter is expressed throughout the oligodendroglial cell cytoplasm. Hence, fluorescence quantification in the selected region reflects the expression of SOX10-EGFP in the cell body and processes. In cultures treated with agathisflavone, it was found that there was no change in SOX10-EGFP expression in the white matter compared to the OGN control, whereas there was a significant reduction in SOX10-EGFP expression in the white matter of cerebellar slices subjected to 1 h of OGD, and this was completely prevented by pretreatment with agathisflavone, which were comparable to OGN slices (Figure 2a,c).

Next, we examined whether these changes in cerebellar white matter were specifically associated with axonal myelination (Figure 2b,d). Myelination of axons was assessed by co-localization of the labeling of myelin basic protein (MBP), a protein constitutive of the myelin sheath, with neurofilament 70 (NF70), a structural protein of neuron axons, using Pearson’s constant. Treatment with agathisflavone did not induce any change in the percentage of MBP+NF70+ myelinated axons (an index of axonal myelination) in OGN. In contrast, it was observed that 1 h of OGD significantly reduced the percentage of myelinated axons (Figure 2d), a reduction of approximately 40%, compared to OGN, which was completely blocked by pretreatment with agathisflavone, which was not significantly different than OGN slices (Figure 2d).

### 2.3. Agathisflavone Prevents the Loss of OPCs in the Molecular Layer in Cerebellar Slices Subjected to Ischemic Damage

**Figure 3:**
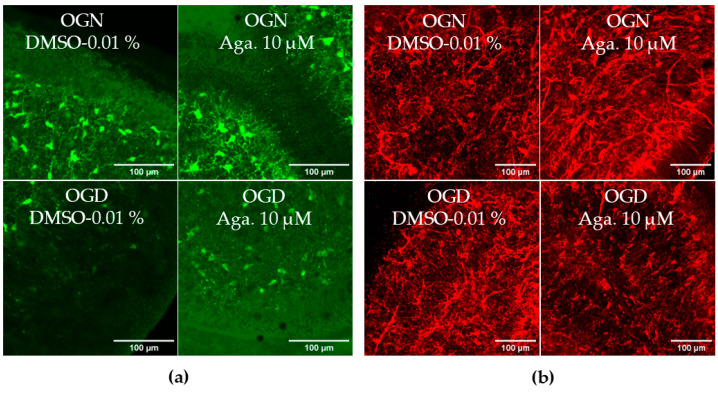

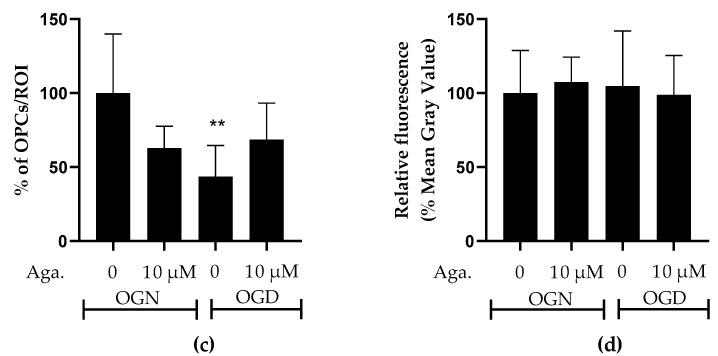
Assessment of the density of oligodendrocyte progenitor cells (OPCs) in the molecular layer and granular layer in cerebellar slices subjected to ischemic damage. (**a**) Organotypic culture of 200 μm thick cerebellar slices in sagittal position from mice aged 8 to 12 d, transgenic with green fluorescent protein reporter of the SOX-10 gene. Images of the molecular layer were obtained using a confocal microscope with a 20× objective and 1.5× zoom. The scale bar represents 100 μm. (**b**) Organotypic culture of cerebellar slices in the sagittal plane, 200 μm thick, from C57BL/6 mice aged between 8 and 12 d. Slices stained by immunofluorescence with anti-NG2. Images obtained under a confocal microscope in the objective at 20× magnification. The scale bar represents 100 μm. (**c**) Bar graphs representing the percentage of the density of OPCs per μm^2^ in the molecular layer in the region of interest (ROI), the percentage was calculated relative to 0.01% OGN-DMSO from the analysis of 2 to 3 images per slice obtained from 3 to 4 cerebellar slices of 3 to 4 animals, and are expressed as the mean and standard deviation; statistical significance was assessed using an analysis of variance ANOVA, *F (3*, *20)* = *4.585*, followed by Tukey’s multiple comparison post-test. The *p*-value is represented by ** *p* < 0.01 compared to the control group (OGN-DMSO 0.01%). (**d**) Bar graphs representing the density of OPCs assessed by immunostaining with anti-NG2 antibody in the granular layer in the constant field of view (FOV) of 425.10 μm × 425.10 μm, the percentage was calculated relative to OGN-DMSO 0.01%, from the analysis of 2 to 3 images per slice obtained from 3 to 4 cerebellar slices of 3 to 4 animals, are expressed as the mean and standard deviation; statistical significance was assessed using an analysis of variance ANOVA, *F (3*, *29)* = *0.1524*.

SOX10-EGFP is expressed by both OPCs and myelinating oligodendrocytes, and to examine whether OPCs were as sensitive to ischemic damage as oligodendrocytes of the white matter and granular layer, we examined SOX10-EGFP+ cells in the molecular layer, which is characterized by not having myelinating oligodendrocytes [28]. Notably, OPCs in the molecular layer showed greater susceptibility to ischemic damage than SOX10-EGFP+ cells in the granule cell layer, which comprise mainly myelinating oligodendrocytes. Agathisflavone treatment in OGN had no significant effect on the density of SOX10-EGFP+ OPCs in the molecular layer but was effective in protecting them in OGD, significantly reducing their loss in this region (Figure 3a).

The effect of flavonoids against ischemic damage in OPCs was also evaluated in the granular layer. For this, we used an antibody specific for the NG2 chondroitin sulfate proteoglycan (NG2), a marker for OPCs (Figure 3b), which generates myelinating oligodendrocytes throughout life [29]. Agathisflavone had no significant effect on the density of NG2+ OPCs in the granular layer, and these cells were not significantly reduced following 1 h of OGD, indicating OPCs may display regional differences in their sensitivity to ischemia (Figure 3b).

### 2.4. Flavonoid Agathisflavone Prevented Astrocyte Reactivity in Acute Ischemic Damage in Cerebellar Slices

**Figure 4:**
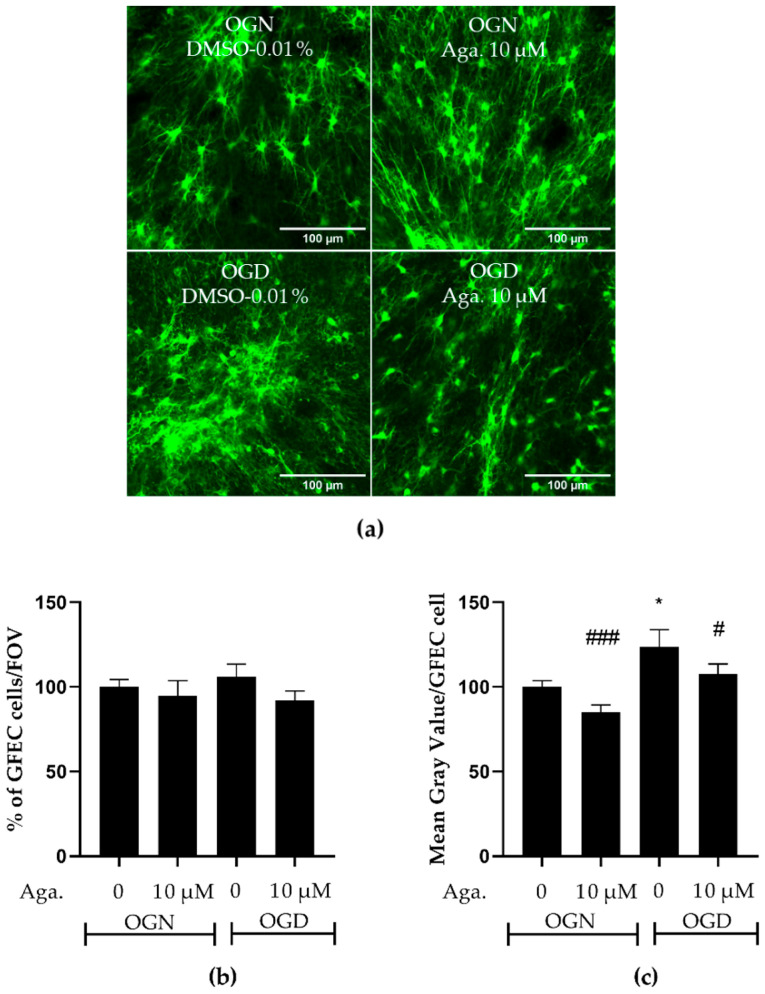
Assessment of astrocyte density and reactivity in cerebellar slices subjected to ischemic damage. Organotypic culture of sagittal slices of cerebellum from GFAP-EGFP (GFEC) mice transgenic, aged between 8 to 12 d, 200 μm thick. (**a**) Images of astrocytes were obtained using a confocal microscope with the objective at 20× magnification and 1.5× zoom. The scale bar represents 100 μm. (**b**) Bar graph representing astrocyte density in the granular layer, quantified in a constant field of view (FOV) of 283.40 μm × 283.40 μm. The percentage was calculated relative to OGN-DMSO 0.01% from the analysis of 2 to 3 images per slice obtained from 3 to 4 slices of 3 to 4 animals and was expressed as the mean and standard deviation and statistical significance was assessed using an analysis of variance ANOVA, *F (3*, *110) = 0.6534*. (**c**) Representative bar graph of GFAP expression per astrocyte in the granular layer, quantified in a FOV of 283.40 μm × 283.40 μm. The percentage was calculated relative to OGN-DMSO 0.01% from the analysis of 2 to 3 images obtained from 3 to 4 slices of 3 to 4 animals and is expressed as the mean and standard deviation; statistical significance was assessed using an analysis of variance ANOVA, *F (3*, *110)* = *6.955*, followed by Tukey’s multiple comparison post-test. The *p*-value is represented by * *p* < 0.05 compared to the control group (OGN-DMSO 0.01%), and # *p* < 0.05 and ### *p* < 0.001 compared to ischemic damage (OGD-DMSO 0.01%).

One common feature of reactive astrocytes is the upregulation of glial fibrillary acidic protein (GFAP), a major protein constituent of astrocyte intermediate filaments. GFAP is upregulated in astrocytes in response to most types of CNS injury and is widely used as a marker of astrocyte reactivity [30,31]. The reactive astrocytes have neurotoxic activity in vitro and were proposed to participate in the pathogenesis of multiple neurologic diseases [30]. The response of astrocytes to ischemic damage when modulated with flavonoids was evaluated in GFAP-EGFP transgenic mice, whose green fluorescent protein is a reporter for the GFAP gene. Both the density of astrocytes and the intensity of GFAP expression, indicative of astrogliosis, were evaluated. It was observed that OGD did not change the density of astrocytes in the granular layer of cerebellar slices, compared to OGN (Figure 4a,b), and pretreatment with agathisflavone did not induce changes in the density of astrocytes in OGN or OGD (Figure 4a,b). In contrast, it was observed that in cerebellum cultures subjected to OGD, compared to OGN controls, there was a significant increase in GFAP-EGFP fluorescence per cell, indicative of astrocyte reactivity, and this was prevented by pretreatment with agathisflavone, which was not significantly different to OGN (Figure 4c).

### 2.5. Agathisflavone Prevented the Loss of Glutamine Synthetase Expression in Ischemic Damage in Cerebellar Slices

**Figure 5:**
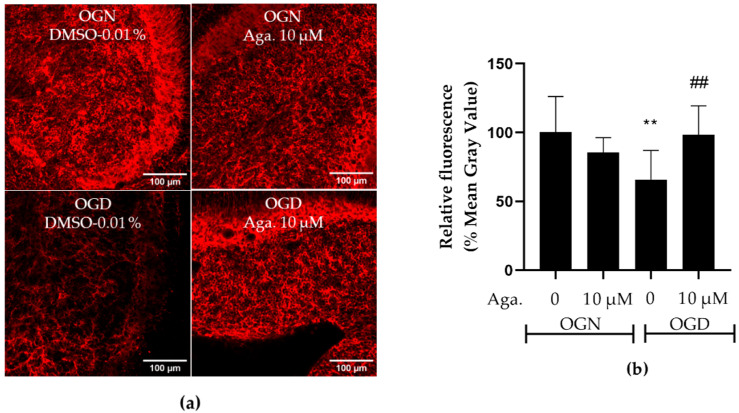
Assessment of glutamine synthetase expression in cerebellar slices subjected to ischemic damage. (**a**) Representative images of GS immunostaining in cerebellar slices in the different treatment groups were obtained using a confocal microscope at 20× magnification. The scale bar represents 100 μm. (**b**) Bar graph of glutamine synthetase enzyme expression in cerebellum slices, quantified in a constant field of view (FOV) of 425.10 μm × 425.10 μm. The percentage was calculated relative to OGN-DMSO 0.01% from the analysis of 2 to 3 images per slice obtained from 3 to 4 slices of 3 to 4 animals and is expressed as the mean and standard deviation; statistical significance was assessed using an analysis of variance ANOVA, *F (3, 34) = 5.854*, followed by Tukey’s multiple comparison post-test. The *p*-value is represented by ** *p* < 0.01 compared to the control group (OGN-DMSO 0.01%), and ## *p* < 0.01 compared to ischemic damage (OGD-DMSO 0.01%).

Astrocytes are involved in glutamate metabolism, regulating the concentration in the extracellular space, to prevent glutamatergic excitotoxicity and consequent neuronal death [8,32], but in ischemic damage, this function is compromised, associated with decreased expression of glutamine synthetase (GS), a key enzyme in glutamate detoxification [33]. Since agathisflavone prevented astrocyte reactivity in OGD, as indicated by increased GFAP-EGFP fluorescence, we examined the expression of GS by immunofluorescence labeling (Figure 5). Agathisflavone had no significant effect on GS expression in OGN, whereas 1 h of OGD significantly reduced GS expression to 65% compared to the OGN control (Figure 5a,b). No reduction in GS expression was observed in cultures subjected to OGD pretreated with agathisflavone (Figure 5b).

### 2.6. Agathisflavone Protects Purkinje Cells from Acute Ischemic Damage

**Figure 6:**
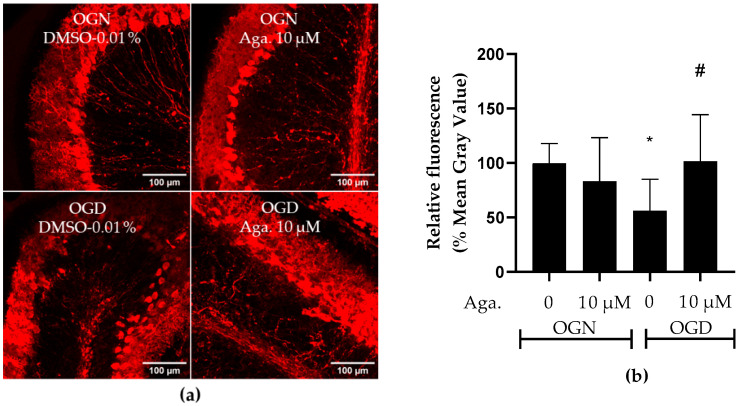
Assessment of the expression of the Purkinje cell biomarker calbindin D-28K in cerebellar sections subjected to ischemic damage. Organotypic culture of cerebellar slices 200 μm thick in the sagittal position of mice aged between 8 and 12 d. (**a**) Images of Purkinje cells obtained under a confocal microscope in the objective at 20× magnification. The scale bar represents 100 μm. (**b**) Representative bar graph of calbindin D 28K expression in cerebellum slices, quantified in the region of interest (ROI). The percentage was calculated relative to OGN-DMSO 0.01% from the analysis of 2 to 3 images per slice obtained from 3 to 4 cerebellar slices of 3 to 4 animals and is expressed as the mean and standard deviation; statistical significance was assessed using an analysis of variance ANOVA, *F (3*, *43) = 4.157*, followed by Tukey’s multiple comparison post-test. The *p*-value is represented by * *p* < 0.05 and compared to the control group (OGN-DMSO 0.01%) and # *p* < 0.05 (OGD-DMSO 0.01%).

Considering that Purkinje cells are neurons particularly vulnerable to ischemic damage [34], the effect of OGD and agathisflavone on Purkinje cells was investigated. To this end, labeling was carried out with antibodies to the protein calbindin D-28K, a protein that binds to Ca^2+^, regulating its homeostasis and playing a crucial role in preventing neuronal death [35]. This protein is considered a specific biomarker for Purkinje cells in the cerebellum [36]. Agathisflavone treatment had no significant effect on calbindin D-28K expression in OGN (Figure 6a,b). Ischemic damage with OGD reduced calbindin D-28K expression in cerebellar slices to approximately 50% (Figure 6a,b), and this was prevented by pretreatment with agathisflavone, which maintained the expression of calbindin D-28K compared to OGN (Figure 6b).

### 2.7. The Expression Profile of Cytokines Related to the Inflammatory Response

**Figure 7:**
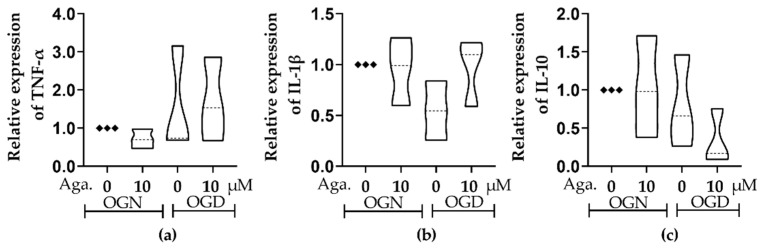
Assessment of the expression of inflammatory and regulatory cytokines in cerebellar sections subjected to ischemic injury. Organotypic culture of cerebellar slices 200 μm thick in sagittal position from mice aged 8 to 12 d, strain C57BL/6. (**a**–**c**) Gene expression of the cytokines TNF-*α*, IL-1*β,* and IL-10, referring to treatment with 10 μM of agathisflavone and respective violin plots. The data are represented in expression relative to the normoxia model (OGN-DMSO 0.01%) and expressed as the median and the interquartile range of results obtained from cerebellar slices of 3 animals. Statistical significance was assessed using an analysis of variance with the Kruskal–Wallis statistical test. Tumor Necrosis Factors Alpha (TNF-*α*), Interleukin 1 Beta (IL1*β*), and Interleukin 10 (IL-10). Samples were analyzed in duplicate. The Glyceraldehyde 3-Phosphate Dehydrogenase (GAPDH) and beta-actin (ACTB) genes were used as endogenous controls for normalization of gene expression data.

Ischemic damage is characterized by an inflammatory response and the consequent production of inflammatory cytokines, namely TNF-*α* and IL-1*β* [7], while lower levels of the regulatory cytokine IL-10 are associated with neurological worsening in patients with acute ischemic damage [37]. The effect of agathisflavone on inflammatory and regulatory cytokines in cerebellar cultures under conditions of OGN and OGD was investigated using RT-qPCR. There were no significant changes in TNF-*α*, IL-1*β,* or IL-10 in OGD compared to OGN controls, and these were not significantly altered by agathisflavone (Figure 7). Nonetheless, there did appear to be some shifts in the IL-1*β* and IL-10 profiles in the different treatment groups, with a possible reduction in IL-1*β* in OGD that was not observed following pretreatment with agathisflavone and slightly reduced IL-10 in OGD which was further reduced in agathisflavone (Figure 7b,c); in contrast, there was no evidence that TNF-*α* was altered by any of the treatments (Figure 7a).

## 3. Discussion

Previous studies have demonstrated that agathisflavone promotes remyelination in the lysolecithin model of demyelination in organotypic cerebellar slices [23,24]. In this study, we show that agathisflavone is protective for oligodendrocytes and myelin against OGD-mediated damage in neonatal mouse cerebellar slices, a model for neonatal stroke.

Oligodendrocytes play a fundamental role in the functional decline observed in several pathologies, such as cerebral palsy, spinal cord injury, and multiple sclerosis [38,39]. In this study, it was observed that ischemic damage in neonatal cerebellar slices did not reduce the density of myelinating oligodendrocytes in the granular layer, which contrasts with evidence in hippocampal cultures, where oligodendrocyte density was decreased after 180 min of OGD [40]. In the present study, it was observed that there was an increase in the number of cells with low processes, which means process retraction, with 60 min of OGD, supporting previous studies on the optic nerve showing that ischemic damage induces the retraction of oligodendrocyte processes within 20 min of OGD [9,40]. It has been demonstrated that this greater susceptibility of oligodendrocyte processes to OGD damage is caused by glutamate excitotoxicity inducing Ca^2+^ influx mediated by NMDA glutamate receptors [9,10]. Oligodendrocytes express ionotropic glutamate receptors unequally [41], with NMDA receptors being distributed in a greater proportion in the processes than in the soma, where there is a greater prevalence for AMPA receptors. Although it is not known whether agathisflavone interacts with NMDA receptors, it has been shown that agathisflavone protects neural tissue from glutamatergic excitotoxicity [33]. More studies are necessary to elucidate if the protection exerted by flavonoids on oligodendrocyte processes may be explained by a possible inhibition of the NMDA receptor, preventing Ca^2+^ influx.

The retraction of oligodendrocyte processes explains the observed decrease in SOX10-EGFP expression in white matter in OGD, an event previously demonstrated in the CA1 region of hippocampal slices from mice subjected to OGD [8]. Agathisflavone was effective in preventing the loss of oligodendroglial processes and myelin in cerebellar white matter. In this study, a higher axonal myelination index determined by Pearson’s coefficient between MBP and NF70 labeling was also observed in cerebellar cultures treated with agathisflavone, compared to OGD damage, in which there was a 40% decrease in myelination index.

In oligodendrocyte progenitors, the expression of AMPA receptor subunits is unequal [42], overexpressing glutamate receptor (GluR) subunits GluR3 and GluR4 but maintaining the expression of the GluR2 subunit, which is responsible for blocking the Ca^2+^ entry, resulting in a greater number of AMPA receptors without the GluR2 subunit, and greater susceptibility of oligodendrocyte progenitors and immature oligodendrocytes to glutamate excitotoxicity [42,43]. This greater susceptibility of OPCs was observed in our study, showing that ischemic damage markedly reduced the density of SOX10-EGFP+ OPCs in the molecular layer, characterized by not having myelinating oligodendrocytes, but did not cause the loss of oligodendrocytes in the granular layer [28]. However, immunolabeling for NG2 did not indicate a loss of OPCs in the granule cell layer, compared to the molecular layer, suggesting regional differences in cerebellar OPCs, such as expressing AMPA receptors with variable Ca^2+^ permeability [44], which requires further investigation to determine whether this explains the resistance to OGD damage of OPCs in the granular layer.

The characteristic response of astrocytes to pathology is termed reactivity, characterized by morphological changes and transcriptomic changes [45]. Astrocyte hypertrophy and increased GFAP labeling are a morphological hallmark of astrocytic reactivity; however, GFAP expression in reactive astrocytes varies significantly depending on their location in the CNS, their proximity to the site of injury, and the type of injury [46]. Astrogliosis is characterized by astrocytic hypertrophy and the consequent release of pro-inflammatory mediators, such as IL-6, TNF-*α*, IL-1*α*, IL-1*β*, and IFNγ, and free radicals, such as NO, superoxide, and peroxynitrite [47]; on the other hand, the glial scar prevents inflammation from spreading to other regions of the nervous tissue [48].

In ischemic injury, astrocytic reactivity is also activated by inflammatory cytokines such as TNF-*α* and IL-1*α* and complement protein C1q secreted by activated microglia; these astrocytes will induce the death of neurons and oligodendrocytes [49]. However, it has already been shown that astrocytes are less susceptible to ischemic damage [50]. In this study, ischemic damage increased the expression of GFAP-EGFP, a marker of astrocytic reactivity. The regulation of astrocyte responses is one of the pathways of action of the flavonoid agathisflavone in neuronal protection after traumatic or inflammatory insult [25,51,52]; it was demonstrated that agathisflavone is effective in protecting cerebellar tissue from astrocytic reactivity.

The level of expression of the GS enzyme, responsible for glutamate detoxification, is also indicative of astrocytic reactivity and plays a crucial role in neurological disorders, including ischemic damage [53]. Although GS has its expression increased in acute ischemia in cerebellar tissue of post-mortem infant patients [54] or after three hours of ischemic damage in rats [55], a decrease in the activity of this enzyme has also already been demonstrated in models of ischemia followed by reperfusion, attributed to the increase in free radicals from reoxygenation [56,57]. In this study, a decrease in GS expression was also observed after 1 h of OGD, with this decrease being prevented by the flavonoid agathisflavone, as demonstrated by Dos Santos Souza and collaborators [33] in co-cultures of neurons and glia. Regarding GS being highly expressed in astrocytes, it can also be expressed in myelinating oligodendrocytes in the granular layer of the cerebellum [58] and in the spinal cord of mice and humans [59], particularly in chronic pathological conditions, such as amyotrophic lateral sclerosis and multiple sclerosis, suggesting that the reduction in GS expression observed in this study may not be solely from an astrocytic source.

Along with granule cells, the Purkinje cells are the major populations of neurons in the cerebellum [60]. Morphologically, cerebellar Purkinje cells have several unique features: the soma are in a thin, single-celled layer between the granular layer and the molecular layer, extending dendrites into the molecular layer, which are arborized into a highly branched structure [61]. Their axons extend from the soma through the granular layer and white matter, projecting to the deep cerebellar nuclei and to a number of target nuclei in the brainstem and the intracortical axons formed by recurrent collateral branches that terminate within the cerebellar cortex [62]. Notably, all computational results within the cortex are transmitted by Purkinje cell axons, which are the neurons that send outputs from the cerebellar cortex, as well as showing several forms of synaptic plasticity [61,62,63]. Cerebellar Purkinje cells are highly sensitive to OGD damage, with glutamate release primarily responsible for the impact on this subpopulation of neurons [64,65]. This type of damage can lead to a reduction in number, changes in cellular morphology, and reduced GABA receptor function, which appears to be related [66]. In addition, it has already been demonstrated that progesterone has a protective role for Purkinje cells [67]. Agathisflavone regulates GABA_A_ receptors [68], facilitating the opening of the GABA-stimulated transmembrane ion channel, which allows an influx of chloride ions, thus resulting in decreased excitability. The protection that agathisflavone conferred on Purkinje cells in the present study against OGD damage could be explained by this interaction with GABA_A_ receptors, although more studies are needed to demonstrate this possibility. Considering that Purkinje cells from young mice do not express functional NMDA receptors [69], it may be that flavonoids act through multiple pathways to protect against ischemic damage in different cell types.

In acute cerebral ischemia, cytokines exacerbate neurological damage, such as TNF-*α* and IL-1*β*, initiating inflammatory reactions [70]. Proinflammatory and anti-inflammatory cytokines are released in the ischemic brain; IL-10 promotes cell survival, and TNF-*α* can induce cell death [71]. The cytokine TNF-*α* exerts different effects in the context of ischemia, showing both neuroprotective and harmful properties, being able to both regulate glutamatergic neurons by reducing excitability and can also contribute to the impairment of the blood-brain barrier and to an increase in the inflammatory response [72,73]. Although, in the acute phase of ischemic damage, this cytokine may have a preventive role in the exacerbation of inflammation in the post-trauma phase [72,74]. After the infarction, the cytokine IL-1*β* is produced by infiltrating microglia and macrophages, compromising the integrity of the blood-brain barrier and allowing the entry of cells from the peripheral immune system [75]. The use of monoclonal antibodies against IL-1*β* [76] in the reperfusion phase after ischemic damage is associated with decreased infiltration of cells from the peripheral immune system [75]. On the contrary, IL-10 is an anti-inflammatory cytokine related to the protection of neural tissue after infarction [77], and it has already been demonstrated that the administration of this cytokine can reduce the inflammatory response after simulated infarction in animal models. In the present study, OGD and flavonoid agathisflavone treatment did not significantly change the expression of either inflammatory TNF and IL-1*β* or regulatory IL-10 after 60 min of ischemic insult, but their modulation after time is not excluded. In fact, in previous studies using glutamate excitotoxicity, the expression of the inflammatory cytokines TNF-*α*, IL-1*β,* and IL 6 were up-regulated and modulated by agathisflavone [33]. Further studies could be developed considering the time of ischemic damage.

## 4. Materials and Methods

### 4.1. Agathisflavone

Agathisflavone was obtained from the extraction of leaves of *Cenostigma pyramidale* (Tul.) as described by Mendes et al. Briefly, dried leaves were individually extracted with MeOH. The crude extracts obtained were partitioned with hexane/MeOH:H_2_O (9:1), CHCl_3_/MeOH:H_2_O (6:4), and after the evaporation of MeOH under vacuum, the aqueous phase obtained was partitioned with EtOAc/H_2_O. The chloroform extract of the leaves was fractionated using column chromatography on silica gel eluted with mixtures of CHCl_3_/EtOAc in increasing polarity; the fraction eluted with CHCl_3_/EtOAc (6:4) followed by gel permeation in Sephadex LH-20 using CHCl_3_/MeOH (2:3) as eluent furnished pure agathisflavone [22,78,79,80]. Agathisflavone was dissolved in DMSO (Sigma, St. Louis, MO, USA) at a stock concentration of 100 mM, stored at a temperature of 4 °C, and protected from light. For experiments, agathisflavone was dissolved fresh at the time of use to make a final concentration of 10 μM in artificial cerebrospinal fluid (aCSF), comprising 133 mM of NaCl, 3 mM of KCl, 2.24 mM of CaCl_2_, 1.1 mM of NaH_2_PO_4_, 1 mM of MgCl_2_, 8.55 mM of HEPES buffer, and 10 mM of glucose, at pH 7.3. The concentration used was selected based on our previous studies on the optimal cytoprotective effect of agathisflavone on oligodendrocytes and myelin in mouse cerebellar slices [23,24].

### 4.2. Animals and Tissue

In this study, male and female mice (*Mus musculus*) were used, between 3 to 4 animals per experiment, aged between postnatal day (P)8–12. Mice were euthanized by exposure to carbon dioxide gas (CO_2_), followed by cervical dislocation and decapitation according to the United Kingdom (Scientific Procedures) Act 1986 (ASPA) on the use of animals and approved by the University of Portsmouth AWERB. A non-transgenic C57BL/6 strain were used, together with transgenic mice, in which the Enhanced Green Fluorescent Protein (EGFP) reporter gene is expressed under the control of the astroglial gene glial fibrillary acidic protein (GFAP) and the oligodendroglial gene transcription factor encoded in HMG-BOX 10 (SOX-10) (gift from Frank Kirchhoff, University of Saarland, Germany and William Richardson, UCL, UK respectively). Brains were removed and placed in ice-cold artificial cerebrospinal fluid (aCSF), comprising 133 mM of NaCl, 3 mM of KCl, 2.24 mM of CaCl_2_, 1.1 mM of NaH_2_PO_4_, 1 mM of MgCl_2_, 8.55 mM of HEPES buffer, and 10 mM of glucose, at pH 7.3.

### 4.3. Ex Vivo Cerebellar Slice Preparation and Oxygen–Glucose Deprivation (OGD)

To examine the effects of agathisflavone preventative treatment on ischemia, cerebellar slices from postnatal SOX10-EGFP mice (P8-12) were used as an ex vivo model that maintains neuron-glial interactions [81,82]. Cerebellar slices were divided into 4 experimental groups, using published protocols [83]: 1, cerebellar slices treated with DMSO (0.01%) vehicle and maintained under normal oxygen and glucose (OGN, aCSF containing glucose and maintained in 95%O_2_/5%CO_2_ at 37 °C); 2, cerebellar slices treated with agathisflavone (10 μM) and maintained under OGN; 3, cerebellar slices treated with DMSO (0.01%) vehicle and maintained under oxygen–glucose deprivation (OGD, aCSF with glucose replaced by sucrose and maintained in 95%N_2_/5%CO_2_ at 37 °C); 4, cerebellar slices treated with agathisflavone (10 μM) and maintained under OGD.

Cerebellar slices were made according to published protocols in our laboratory [84]. In brief, the cerebellum was mounted on a vibratome (Camden Instruments LTD, Leicestershire, UK), and sections were cut in the sagittal plane at a thickness of 200 μm in ice-cold aCSF. Cerebellar slices were collected in 6-well plates in aCSF and randomly selected for pretreatment with the flavonoid agathisflavone (10 μM) or with the vehicle DMSO 0.01% (control) and then incubated under OGN (95%O_2_/5%CO_2_) conditions at 37 °C for 60 min. After this period, the incubation solution was replaced with fresh aCSF containing agathisflavone or DMSO vehicle, as appropriate, and slices were randomly selected for OGN or OGD conditions. For OGD, slices were incubated in aCSF in which glucose was replaced by sucrose and transferred to a hypoxia chamber (95%N_2_/5%CO_2_) at 37 °C (Figure 1c). Slices were maintained in OGN or OGD for 60 min, after which they were washed with PBS and either immersion fixed in 4% paraformaldehyde for subsequent immunofluorescence analysis or were immersed in RNALater Qiagen (Hilden, Germany) solution for subsequent RT-qPCR analysis.

### 4.4. Immunofluorescence Labeling

After 1 h of fixation, slices were washed with PBS (3 × 10 min) and preserved in phosphate-buffered saline (PBS) at 4 °C for subsequent immunolabelling. Slices were permeabilized with 1% Triton x-100 in PBS overnight at 4 °C. Non-specific binding was then blocked with 20% bovine serum albumin (BSA) in PBS-T (0.01% Triton x-100 in PBS) for 3 h in continuous rotation. The blocking solution was removed and the slices were incubated with the primary antibodies mouse anti-myelin basic protein (MBP) (1:300, MAB 386, Burlington, MA, USA), mouse anti-neurofilament 70 (NF70) (1:300, MAB 1615, Burlington, MA, USA), rabbit anti-chondroitin sulfate (NG2) proteoglycan (1:250, Millipore AB5320, Darmstadt, Germany), mouse anti-calbindin D-28K (CB) (1:1000, Swant CB300PUR, Burgdorf, Switzerland), or rabbit anti-glutamine synthetase (GS) (Abcam ab49873, Darmstadt, Germany), dissolved in PBS-T with 1% normal goat serum (NGS) overnight at 4 °C. The primary antibody solution was removed, and the slices were washed in PBS-T (3 × 10 min) in continuous rotation. Secondary antibodies were goat anti-mouse (1:1000, Alexa Fluor 647) (Invitrogen A21247, Waltham, MA, USA), goat anti-mouse (1:1000, Alexa Fluor 488) (Invitrogen A11001, Waltham, MA, USA), goat anti-mouse (1:1000, Alexa Fluor 647) (Invitrogen A21235, Waltham, MA, USA) or goat anti-rabbit (1:1000, Alexa Fluor 647) (Invitrogen A21244, Waltham, MA, USA), diluted in PBS-T with 1% NGS in the presence of the nuclear dye Hoechst 33342, trihydrochloride trihydrate (5 μg/mL) (Invitrogen, H1399, Waltham, MA, USA) for 3 h in continuous rotation. At the end of this last incubation, the slices were washed with PBS-T (3 × 10 min) and then with PBS (5 × 5 min). Slices were mounted in VectaMount^®^ permanent mounting medium (H-5000-60, Newark, CA, USA).

### 4.5. Image Acquisition and Analysis

Images were acquired using a Zeiss (Oberkochen, Germany) LSM 710 confocal microscope in z-stack planes 10 µm apart, and acquisition parameters were maintained between each experiment. Three to four photographs were taken per slice, with three to four slices per condition. Images were captured using a ×20 objective and obtained from the cerebellar lobes, the granular layer, the molecular layer, and the white matter; all slices were imaged, and all images were included in the analyses. The relative fluorescence intensities of SOX10-EGFP, GFAP-EGFP, and immunostaining for NG2, MBP, NF70, GS, and CB were measured using Fiji-ImageJ version 2.14.0. The measurement of the density of oligodendrocytes and astrocytes in the granular layer was determined by counting the number of SOX10-EGFP+ or GFAP-EGFP+ cells, respectively, in a constant field of view (FOV) of 286.43 μm × 286.43 μm, applying a binary watershed filter and counting particles with an area between 20 and 200 μm^2^, using Fiji-ImageJ version 2.14.0. The morphology of oligodendrocytes was evaluated in SOX10-EGFP+ cells, applying a convolution filter with the following matrix, = [−1 −1 −1 −1 −1\−1 −1 −1 −1 −1\−1 −1 24 −1 −1\−1 −1 −1 −1 −1\−1 −1 −1 −1 −1\], and applying a binary skeletonize filter, proceeding with the analysis in the Analyze Skeleton Plugin (2D/3D). In the images generated, a grid was drawn with squares of 5000 μm^2^, and quantification was carried out in 5 of these squares as shown in Figure 1c (areas highlighted in green), where the number of processes in each cell was quantified, and cells were categorized according to the number of processes per cell, as low (<5), medium (5–9) and many (>9). White matter was analyzed by quantifying SOX10-EGFP fluorescence in the region of interest (ROI) delimited to the white matter in cerebellum slices.

### 4.6. Quantitative RT-PCR

Total RNA was extracted with Trizol^®^ reagent from slices previously reserved in RNALater Qiagen (Invitrogen, Waltham, MA, USA, Life Technologies, 15596026). The extraction was carried out according to the manufacturer’s specifications. Total RNA purity and concentration were determined by spectrophotometric analysis using KASVI Nano Spectrum (cat# K23-0002). DNA contaminants were removed by treating the RNA samples with DNase using the Ambion DNA-free kit (cat# AM1906, Life Technologies™, Carlsbad, CA, USA). For cDNA synthesis, SuperScript^®^ VILO™MasterMix (cat# MAN0004286, Invitrogen™, Life Technologies) was used in a 20 μL container reaction with a concentration of 2.5 μg of total RNA, following the manufacturer’s instructions. The quantitative real-time PCR (RT-qPCR) was performed using Taqman^®^ Gene Expression Assays (Applied Biosystems, Carlsbad, CA, USA) containing two primers to amplify the sequence of interest, a specific Taqman^®^ MGB probe and TaqMan Universal Master Mix II with UNG (cat# 4440038 Invitrogen, Life Technologies™). The assays corresponding to the genes quantified in this study were Tumor Necrosis Factors Alpha (TNF-*α*), Interleukin 1 Beta (IL1*β*), and Interleukin 10 (IL-10). Samples were analyzed in duplicate. The Glyceraldehyde 3-Phosphate Dehydrogenase (GAPDH) and Beta-Actin (ACTB) genes (Table 1) were used as endogenous controls for the normalization of gene expression data. Real-time PCR was performed using the Quant Studio 7 Flex™Real Time PCR system (Applied Biosystems, CA, USA). Thermocycling conditions were carried out in accordance with the manufacturer’s specifications. Data were analyzed using the 2-ΔΔCt method. The results represent the median of three experiments.

### 4.7. Statistical Analysis

The statistical analyses and respective graphs were generated in GraphPad Prism 8 software. Firstly, it was assessed whether the data presented a Gaussian distribution with the Shapiro–Wilk and Kolmogorov–Smirnov tests, assuming a Gaussian distribution when both statistical tests presented a value of *p* ≥ 0.05 in all variables, and thus proceeding to test the hypotheses with parametric tests; otherwise, the hypotheses were tested with non-parametric tests. For samples with Gaussian distribution, an analysis of variance (ANOVA) was performed, followed by Tukey’s post-test which compared the different variables. For samples with non-Gaussian distribution, an analysis of variance was performed with the Kruskal–Wallis non-parametric test, followed by Dunn’s multiple comparison test. The categorization of cells according to the number of processes was carried out in a contingency table, and the distributions of the respective variables were compared with the Chi-square test. Confidence intervals were defined at a 95% confidence level (*p* < 0.05 was considered statistically significant).

## 5. Conclusions

The neuroprotective and glial-modulating effects of the flavonoid agathisflavone have been demonstrated in different models of neuropathology, including injury and demyelination. In the present study, we demonstrate that preventative administration of agathisflavone in neonatal mouse cerebellar slices inhibits ischemia-induced loss of Purkinje neurons and protects oligodendroglial cells from atrophy, preserving their morphology and axon myelinating capacity. Furthermore, we show that astrogliosis induced by ischemia is reduced by agathisflavone pretreatment, which notably maintains astroglial expression of glutamine synthetase, an essential enzyme for the detoxification of glutamate, which plays a key role in ischemic cytotoxicity. The results support the potential use of agathisflavone preventive treatment in protecting neural cells from ischemic damage, which is relevant to neonatal stroke, traumatic injury, and other neuropathologies.

## Figures and Tables

**Table 1:** Genes used in gene expression analysis and their respective NCBI identification codes (ID) and the forward and reverse sequences of the respective primers.

Gene	Gene ID	Direct Sequence	Reverse Sequence
TNF-*α*	21926	GGTGCCTATGTCTCAGCCTCTT	GCCATAGAACTGATGAGAGGGAG
IL-1*β*	16176	TGGACCTTCCAGGATGAGGACA	GTTCATCTCGGAGCCTGTAGTG
IL-10	16153	CGGGAAGACAATAACTGCACCC	CGGTTAGCAGTATGTTGTCCAGC
GAPDH	14433	CATCACTGCCACCCAGAAGACTG	ATGCCAGTGAGCTTCCCGTTCAG
ACTB	11461	CATTGCTGACAGGATGCAGAAGG	TGCTGGAAGGTGGACAGTGAGG

## Data Availability

Data are contained within the article.
